# Odontological Society of Pennsylvania

**Published:** 1892-02

**Authors:** 


### ODONTOLOGICAL SOCIETY OF PENNSYLVANIA.

*December 12, 1891.*

### INCIDENTS OF PRACTICE.

*Dr. Darby.*—I have in this bottle an interesting growth which was recently removed from the mouth of a gentleman. Its position was the roof of the mouth, under a badly-fitting rubber plate. You will observe that it is an inch in length, seven-eighths of an inch in width, and more than a quarter of an inch in thickness. When the gentleman removed his plate preparatory to having some fillings inserted, I observed this growth, which resembled the hypertrophy of mucous membranes often seen where a badly-fitting plate, having a vacuum, is worn. A more careful examination revealed the fact that the mass was loose, and only attached to the surrounding tissue by a small pellicle not greater in diameter than a small quill.

Its removal was a simple matter, which was accomplished with a sharp bistoury. The hemorrhage which followed was profuse. I am sure I speak within bounds when I tell you that nearly a quart of blood was lost before the flow could be arrested.

Upon inquiry, I learned that he had worn a rubber plate for twenty years. This plate supported four incisors, and had bicuspids on either side. It had a large vacuum-chamber, and the superficial dimensions of the tumor corresponded with the shape of the chamber.

The peculiarities of the case are these: First, the enormous size which it had attained; second, the fact that it was loose and entirely detached except as above mentioned; and third, the healthy condition of the roof of the mouth immediately under the growth.

Dr. Gaskill said that he had had a similar case to that described by Dr. Darby, which he preferred to ligate in preference to excision. This was accomplished without the slightest hemorrhage. It covered a space fully equal to the diameter of a dime. It was tied with a silk ligature, and the portion removed was nearly a quarter of an inch thick.

Dr. Truman stated that he thought it was a case of non-malignant epithelioma, saying, "I have a case very similar,—a tumor that has been on the alveolar border for a number of years. I advised that it be let alone or else taken off with a ligature, which the patient objected to having done."

Dr. Peeso was then introduced, and read his paper on Crown- and Bridge-Work.

(For Dr. Peeso's paper, see page 90.)

*Dr. Darby.*—I have been very much pleased while listening to that paper and in the examination of the work before it was read.

It is not often that we see such beautiful operations. If I mistake not, all of the nine teeth shown in the superior jaw have bands around the roots, and yet none of these bands are visible from the front view, and there did not seem to be the least irritation of the gums about the teeth. To accomplish such results is no mean task, and you will agree with me that it shows great skill and accuracy of detail.

So far as bridge-work itself is concerned I have not had great experience. I have inserted quite a number of small pieces, and they have proven very satisfactory. I have seen a great many unsatisfactory ones, often the result of bad judgment and worse workmanship. There is no doubt but that much of the bridgework is of poor mechanical construction. When it has been nicely done the testimony of those who wear it is strongly in its favor. I recently saw an extensive piece of this kind of work. There were at least ten or twelve teeth attached to three or four crowns and roots. It had been in use several years, and was in every way satisfactory to the wearer.

Dr. Rymer, of England, was called upon, who said, "I fear in England we are very apt to run down bridge-work, and I think the reason is that we don't understand the way to make it. I am satisfied very few have been trained in the proper kind, but I see no reason why it should not be a success. I hope before I return to England to take with me some few hints, and I shall always have a pleasant remembrance of my first meeting with the Odontological Society of Pennsylvania."

*Dr. Head.*—May not a cantilever bridge often be more serviceable than one fixed at both ends? I have in mind two cases which may illustrate my meaning. In the first a lateral was to be inserted between a perfectly firm canine and central. The patient was anxious to avoid a plate. A half crown was fitted to the canine. A lateral, with a gold spur to rest against the palatine surface of the central, was attached. Thus in mastication the movement of the teeth did not tend to loosen the foundation of the structure, and yet all blows were met by efficient resistance.

In the other case, a second lower bicuspid was to be replaced. The lower sixth-year molar was covered with a Richmond crown, to which was soldered a gold second bicuspid. From this gold tooth a spur rested on a Richmond crown, previously placed on the first bicuspid. This has proved to be not only strong but cleanly, as a thread could be freely used, and yet the downward pressure absolutely sustained.

*Dr. Peeso.*—I have seen a great many cases where they have been cemented in or filled with gold, and unless they are strongly dovetailed they are almost sure to work loose. They should be so that a thread can be passed under them and thus kept clean.

*Dr. Jefferies.*—I have not had a great deal of experience with bridge-work. I was reminded of a case similar to the one Dr. Head mentioned, which I put in a little over a year ago. It was the superior deciduous canine, lost by absorption of the root, and, as the patient had never cut her second canine, I suspended it to a first bicuspid, which happened to be short and didn't require a very large or conspicuous gold cap; it has a simple spur on the other edge, resting on the palatal surface of the lateral incisor, and it looks well and is perfectly firm and satisfactory.

My experience with bridge-work has been exceptionally favorable. I never put in a piece unless I am perfectly satisfied it will be permanent.

*Dr. Kirk.*—We have certainly had shown to us this evening that there are two kinds of bridge-work,—good and bad,—and particularly what is meant by the former. There are two questions that have arisen in my mind. In the first place, the question of repairing. Then the other point I want to ask about is this: What limitations does the essayist place upon the insertion of bridge-work? For example, I have been unfortunate enough to lose several teeth,—two bicuspids and one sixth-year molar on one side, and one bicuspid and a sixth-year molar on the other. Now, the teeth he would necessarily select as abutments for a bridge in my case are absolutely sound. In such a case as described, would he consider the insertion of bridge-work a proper thing to do, or would he advise me to wear a plate or go without, which I am now doing?

*Dr. Peeso.*—There are different ways of doing it. It would take too long to go into the details of the work.

*Dr. Kirk.*—Suppose between the abutments of a permanent bridge you have some three or four crowns, and one breaks, what method do you pursue in repairing?

*Dr. Peeso.*—I have never had to repair a piece in the posterior portion of the mouth. I do not make provision for possible breakage. The general practice (illustrating on board) is to drill and let the pin extend through, and, placing cement in between the face and the gold, rivet it in. I use a plate punch that you can apply readily over the tooth and grip with the point of the pliers and rivet by pressure. I don't think there is any danger of the face breaking off the cap. I have never had a facing break but in one lateral incisor, that I replaced two or three weeks ago where it had been broken off.

Dr. Truman inquired if that riveting process would hold, to which Dr. Peeso answered that it could be riveted so as to be quite firm by drilling holes and taking the entire strain off the face.

Dr. Head asked if it is not sometimes advisable not to put on a facing, but to have it all gold.

*Dr. Peeso.*—It would make it unsightly to have a strip of gold. The thin veneer of porcelain takes very little space, and leaves a solid triangular piece of gold which will give all the strength required.

*Dr. Kirk.*—I have had in two or three instances good results by riveting with the Bonwill mallet. I have been able by heavy blows to put on veneers very tightly by using the Bonwill mechanical mallet with a convex smooth point instead of a hammer or riveting punch.

*Dr. Place.*—I have in mind a young man who has all the molars missing on one side except the wisdom tooth, and that is perfectly sound; the first bicuspid is also perfectly sound, and the canine is missing. Would you devitalize the nerve of the bicuspid to put on bridge-work, or would you leave it as it is, or how would you arrange it?

*Dr. Peeso.*—This is a question very hard to decide. It would depend a great deal whether the tooth is very prominent or whether it shows very plainly. It is impossible to decide without seeing the mouth. It could be done by putting a full gold cap on the first bicuspid. Placing six teeth on two roots would make a pretty severe strain, but in a great many cases where the teeth are perfectly solid and firm, the case would last for a good many years.

I don't claim to be an expert, and do not feel capable of answering all questions, but during the time I have been engaged in the work I have given it careful study.

*Dr. Darby.*—Dr. Bonwill has just called my attention to a peculiar case of bridge-work which I showed him last spring. Two molars and a bicuspid had been lost from the inferior jaw. The teeth of the superior were all present and good. It was thought advisable to put something in this space, but a grave difficulty presented. The wisdom tooth, which was to form one of the abutments, had tipped very much forward, but was firmly fixed in the jaw. The first bicuspid, which was to be capped and form the other abutment, had tipped backward. It will therefore be seen that inasmuch as the space near the gum was much larger than between the ends of the teeth, it would be impossible to put the bridge in place if made in one piece. It was therefore decided to make the teeth of gold (hollow gold crowns). This was done, and, when finished, the piece was divided between the caps covering the bicuspid and molar crown, leaving those two intact. The three hollow crowns were then filled with rubber and vulcanized. Two large screws were then put through gold, rubber, and all. The pieces were inserted separately, and when the cement was in the act of setting, the two screws were driven home, bringing the two halves of the bridge nicely together. The result was eminently satisfactory, and I cannot see how it could have been done in any other way.

*Dr. Bonwill.*—I look at the case of the essayist with a great deal of pleasure. To my mind it was evidently done very thoroughly, and it looks also, judging from the details given, that it should be very permanent so far as bridge-work can be permanent. Dr. Peeso has covered the ground very nicely, and, what is better, brings up many points against bridge-work. He is not willing to advocate it in every case; I don't think he would be foolish enough to attempt it in a good many I have seen. He may grow bolder as he goes on in the work, and probably overstep the bounds, as a great many others have done. Only one man out of a great many can do it.

I really see but one mistake in this gentleman's work, and that is in the articulation. Without proper articulation it is of little use to the patient. If he will take his case and put the lower jaw in lateral movement on either side, he will find there is no proper articulation. This effect you can see here (indicating) without difficulty. The only thing I would say to the essayist is, learn to articulate properly. Had this been done, the case he has brought here would have been of seventy-five per cent. greater value to the patient. It is impossible to articulate teeth as he has arranged them and get lateral movements.

I have never banded a tooth since 1873, when I commenced to put on crowns, cementing them with the best oxyphosphate. American teeth are not suitable for this work. Now that I use English crowns I have no trouble.

I have brought some casts with me to ask the essayist and those here what they would do under the circumstances. What teeth they would mutilate and from which they would remove the living pulps. They are extreme cases, and I wish your verdict upon them.

It will soon be necessary that dentistry be divided into specialties, and that dentists having cases and recognizing that some other one can do the work in a way that will be much better for the patient should send the patient to such a person, and not presume to do it in an inferior way. All dentists should be willing to confess that in some matters there are others who can perform the work in a better manner than they can.

*Dr. Truman.*—I know comparatively little practically in regard to bridge-work. I am on record in this Association as in opposition to it and crowns, and I think we have had evidence sufficient to warrant opposition to at least unskilled work.

The first impression I received from the original Richmond crown was that a band placed around a tooth would certainly arouse pericemental inflammation, and that it could not be otherwise so long as it pressed on the pericementum, and I have never wholly abandoned that opinion. Therefore it has been a satisfaction to see this case so free from inflammatory conditions, and causes me at least to modify my former expressed views.

As Dr. Bonwill has stated, there is bridge-work and bridge-work. I question the general ability in the dental profession today to do this. I doubt whether it be possible for many who attempt it to place a band around a tooth that will not arouse inflammation. If the bands fail to fit the roots, inflammation is certain to be developed. It is because the essayist has placed those bands so thoroughly and so perfectly that fluids cannot pass in between them that he has avoided that difficulty. Now, who is capable of doing that? It requires a skilled mechanician. It requires more than this, a man capable of comprehending possible pathological conditions that are likely to supervene, and to understand exactly what he has to do to overcome them when they arise. The time has come for a division of dentistry into special work. There are some of us not capable of doing certain things, and we might as well know it and acknowledge it at once, and not undertake to do everything, oftentimes to the injury of patients.

Adjourned.

